# Mid-upper arm circumference as an indicator of underweight in adults: a cross-sectional study from Nepal

**DOI:** 10.1186/s12889-020-09294-0

**Published:** 2020-07-29

**Authors:** Lene Thorup, Sophie Amalie Hamann, Per Kallestrup, Vibeke Elisabeth Hjortdal, Ashish Tripathee, Dinesh Neupane, Cecilie Blenstrup Patsche

**Affiliations:** 1 Department of Cardiothoracic & Vascular Surgery, Aarhus University Hospital, Palle Juul-Jensens Boulevard 99, 8200 Skejby, Aarhus N Denmark; 2 Center for Global Health (GloHAU), Department of Public Health, Aarhus University, Aarhus, Denmark; 3 Department of Clinical Medicine, Aarhus University, Aarhus, Denmark; 4 Western Regional Hospital, Pokhara Academy of Health Sciences, Pokhara, Nepal; 5 Department of Epidemiology, Welch Center for Prevention, Epidemiology, and Clinical Research Johns Hopkins Bloomberg School of Public Health, Baltimore, USA; 6 Nepal Development Society, Bharatpur, Chitwan Nepal

**Keywords:** Anthropometry, Body mass index, Epidemiology, Mid-upper arm circumference, Public health, Nutrition, Tropical medicine

## Abstract

**Background:**

Undernourished people have an increased risk of premature mortality from both infectious and non-communicable diseases. Aside from screening purposes, assessment of nutritional status is a useful tool in management and evaluation of various chronic diseases. Body-Mass-Index (BMI) is today the most commonly used marker of nutritional status however, this method presents a challenge in many low resource settings and immobile patients. Mid-upper arm circumference (MUAC) is another anthropometric measure that requires minimal equipment and little training. So far, MUAC cutoffs for undernutrition are well established in children < 5 years but there is still no consensus for a specific cutoff in adults. The objective of this study was to compare MUAC with BMI and suggest a MUAC cut-off corresponding to a BMI of 18.5 kg/m2 to identify underweight in adults.

**Methods:**

A cross-sectional study was conducted at two urban public hospitals in Nepal. The following variables where collected: MUAC, weight, height, sex, age and self-reported medical history. Exclusion criteria: < 19 years of age, pregnancy and oedema. Sensitivity and specificity for a MUAC value corresponding to BMI < 18.5 was calculated. ROC analysis was performed for male and female as well as Pearson’s correlation of MUAC and BMI.

**Results:**

A total of 302 people between 18 and 86 years of age, 197 women and 105 men, were included. Of these, 90 people suffered from rheumatic heart disease. MUAC was highly correlated with BMI in both women *r* = 0.889 and men *r* = 0.846. Best statistically derived MUAC cutoff corresponding to a BMI < 18.5 kg/m^2^ was 24.5 cm (Youdens Index = 0.75; sensitivity 92.86; specificity 82.48), with high predictive value (AUROCC> 0.9). The setting based optimal MUAC cutoff was also 24.5 cm. No considerable variation was found in sex- and disease specific subgroups.

**Conclusion:**

MUAC is strongly correlated with BMI in adults in Nepal. For simplicity, a MUAC of 24.5 cm is the optimal statistically and setting based cutoff in both women and men to identify underweight (BMI < 18.5 kg/m^2^).

## Background

For over two decades, the World Health Organization has recommended body mass index (BMI) to assess nutritional status in adults [1], despite difficulties assessing fat-free mass In chronic undernutrition, the body loses both fat mass and fat-free mass. Since muscle tissue constitutes large parts of the fat-free mass loss [2], anthropometric measurements that take into account muscle atrophy such as mid-upper arm circumference (MUAC), could be a better alternative to BMI. Not only is BMI challenged when used as an indicator of chronic undernutrition [3], MUAC also outperforms BMI when comparing the ability to predict all-cause mortality [4, 5]. Despite seemingly simple, determining BMI can pose a challenge, especially in low-resource settings. Good-quality equipment is expensive, needs calibration and requires trained literate manpower. Furthermore, BMI, no matter the setting, often is difficult to perform on very ill and immobile patients.

Measuring MUAC is a low-cost procedure, requires minimal equipment and maintenance and can be measured by illiterate people. However, globally recognized recommended cutoffs are only available for detecting acute malnutrition in children aged 6–59 months [6]. Despite this, MUAC is already being used as a screening tool for nutritional status in adults in various human immunodeficiency virus (HIV) and tuberculosis health programs [7]. The correlation between BMI and MUAC and MUAC cutoffs identifying underweight in assumed healthy adults have been suggested in numerous studies [8]. Yet, no official, globally recognized MUAC cutoffs have been established for adults and we do not know if there is a difference between men and women. To the best of our knowledge, no studies have examined the association between MUAC and BMI in a population including chronically ill patients except from HIV-infected populations.

Therefore, the present study sought to assess MUAC as an alternative to BMI to detect adult underweight in a population, which also includes chronically ill patients, in this case suffering from rheumatic heart disease. The aim was to suggest an appropriate MUAC cutoff for the equivalent of a BMI < 18.5 kg/m^2^ (BMI cut off for underweight), compare it with cutoffs identified in healthy populations and investigate possible sex related differences in the proposed cutoff.

## Methods

### Study design, setting and participants

The present cross-sectional study was derived from a case-control study on rheumatic heart disease (RHD) examining the relationship between Vitamin D deficiency and RHD (unpublished observations, submitted, principal investigator Lene Thorup). The study was conducted from March to July 2018 in Nepal. Patients with rheumatic heart disease were recruited from the two largest public health facilities receiving cardiac patients in Nepal; Department of Cardiology at Western Regional Hospital in Pokhara and Manmohan Cardiothoracic Vascular and Transplant Center in Kathmandu. Both hospitals provide health services for everyone regardless of social class and ethnicity. Controls were recruited from the same hospitals among relatives/accompanies, and, in Pokhara, additional participants for the present study were sampled from the wound/dressing clinic. For the original case-control study, 202 people were screened for inclusion (105 patients with RHD and 97 cardiac healthy controls), and an additional 154 participants were screened for inclusion for the present cross-sectional study. Exclusion criteria for all participants were age < 18 years, pregnant women, and the presence of edema.

### Anthropometry

Anthropometric measurements were performed following WHO standards by the same investigator using the same equipment throughout the study. Participants were weighed on a digital scale to nearest 0.1 kg (kg) and height was measured in cm to nearest 1 mm. Before this, participants were asked to remove bags, shoes and other heavy clothing and items. MUAC was measured with a non-stretchable MUAC-tape (UNICEF, Adult, S0145630) on the participant’s relaxed left arm at the midpoint between the olecranon and acromion. BMI was calculated as weight divided by height squared (kg/m^2^).

Information about age, sex and previous history of disease and current comorbidities were also collected based on self-report and by examination of the participant’s record book if available.

### Statistical analysis

Data were collected in hard copies and entered with REDCap electronic data capture tools. Statistical analyses were performed using Stata Statistical Software IC 15.1 (StataCorp LP, TX). Normality in distribution was tested using Q-Q plots. Scatterplots with fitted linear regression lines were calculated to assess the relationship between MUAC and BMI, and subsequent correlation analysis was performed using Pearson’s correlation. Sensitivity and specificity were calculated for all individual measurements in the dataset. Youden’s Index (YI) was calculated as YI = sensitivity+specificity-1. The MUAC cutoff with the highest YI-value was considered the optimal statistically-derived cutoff [9]. Receiver-operating characteristic (ROC) curves were calculated for all participants and women and men separately. The area under the ROC curve (AUROCC) was used as the accuracy of the ROC test. Furthermore, 2 × 2 tables were created for MUAC cutoffs at every 0.5 cm and sensitivity (SENS), specificity (SPEC), positive predictive value (PPV) and negative predictive value (NPV) were calculated. Throughout, low BMI/underweight was defined as BMI < 18.5 kg/m^2^.

## Results

A total of 356 participants were screened for inclusion, 216 from Pokhara and 140 from Kathmandu. Of these, 105 suffered from RHD. Subsequently, 54 were excluded; 26 were underage, 9 had edema, 16 were pregnant and 3 had missing data for analysis. This yielded a total of 302 participants of which 90 had RHD. Anthropometric values and descriptive characteristics of participants are presented in Table 1.

**Table 1:** Descriptive characteristics of all participants

Characteristics	***N*** = 302
**Age (years)**	
Mean ± SD	37.8 ± 14
Min - Max	18–86
Median (25th, 75th)	34 (27, 46)
**Sex**	
Female	197 (65.2%)
Male	105 (34.8%)
**RHD status**	
RHD-positive	90 (29.8%)
RHD-negative	212 (70.2%)
**MUAC (cm)**	
Mean ± SD	27.0 ± 3.4
Min - Max	19.5–41
Median (25th, 75th)	26.7 (24.5–29.1)
**Height (cm)**	
Mean ± SD	156.6 ± 8.5
Min - Max	139.6–180.2
Median (25th, 75th)	155.3 (150, 163)
**Weight (kg)**	
Mean ± SD	59.6 ± 12
Min - Max	33.8–106.1
Median (25th, 75th)	58.2 (51.3–66.5)
**BMI (kg/m**^**2**^**)**	
Mean ± SD	24.2 ± 4.6
Min - Max	15.1–44.7
Median (25th, 75th)	23.7 (20.8–27.4)
**BMI categories**	
< 18.5	28 (9.3%)
18.5–25.0	158 (52.3%)
25.0–30.0	83 (27.5%)
≥ 30.0	33 (10.9%)

In total, 9.3% of the participants had low BMI < 18.5 kg/m^2^, and the majority had BMI values within the normal range (18.5 kg/m^2^–25 kg/m^2^). Overweight (BMI 25 kg/m^2^–30 kg/m^2^) was highly prevalent with 27.5, and 11% were obese (BMI > 30 kg/m^2^).

**Fig. 1:**
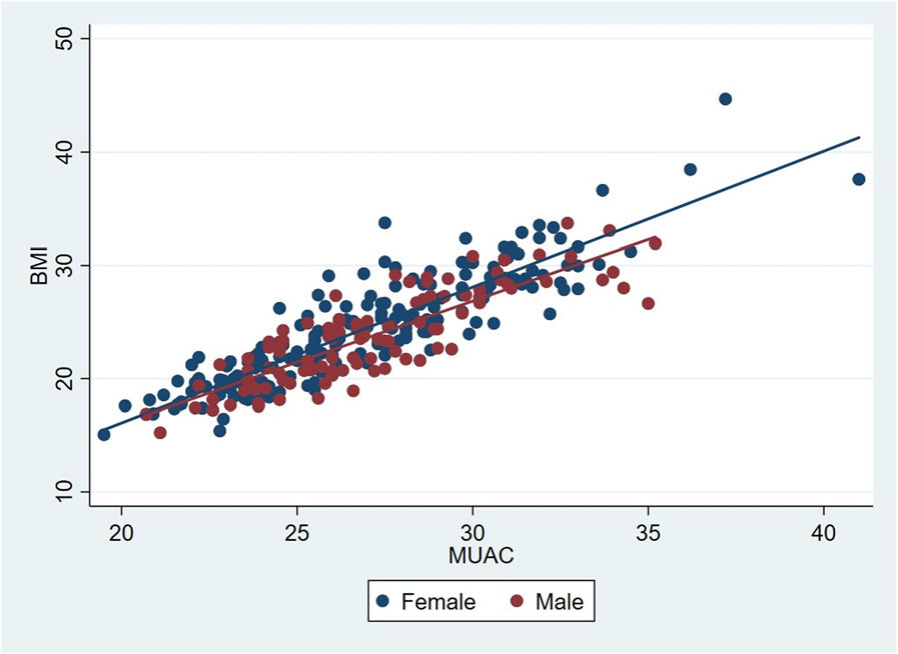
Scatterplot of BMI by MUAC, divided by sex

A scatterplot of BMI by MUAC with regression fits for women and men is illustrated in Fig. 1. There was no difference between men and women. MUAC and BMI were highly correlated, demonstrated by Pearson’s correlation coefficient of 0.872 combined (0.889 for women and 0.846 for men).

**Fig. 2:**
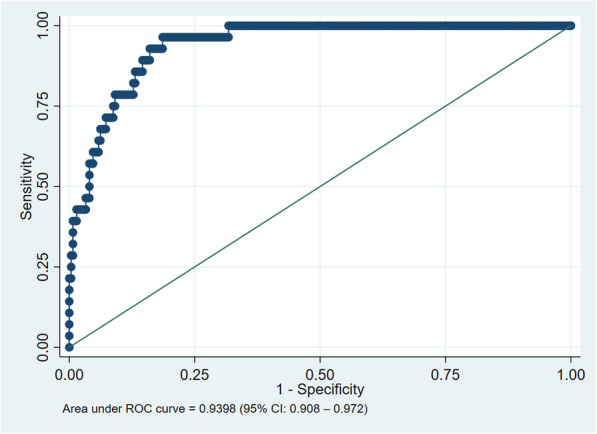
Reciever operating characteristics curve of mid-upper arm circumference based on BMI < 18.5 kg/m^2^, all participants

**Fig. 3:**
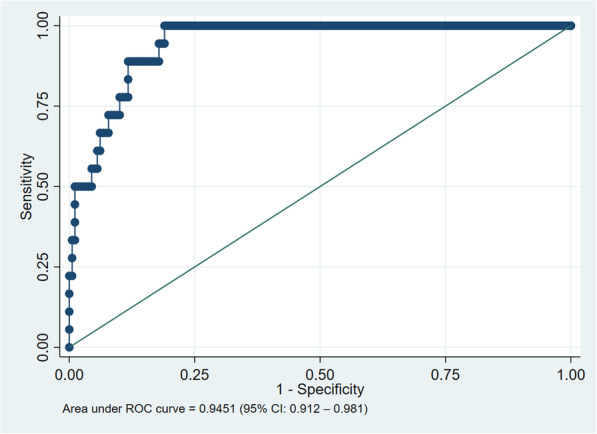
Reciever operating characteristics curve of mid-upper arm circumference based on BMI < 18.5 kg/m^2^, only females

**Fig. 4:**
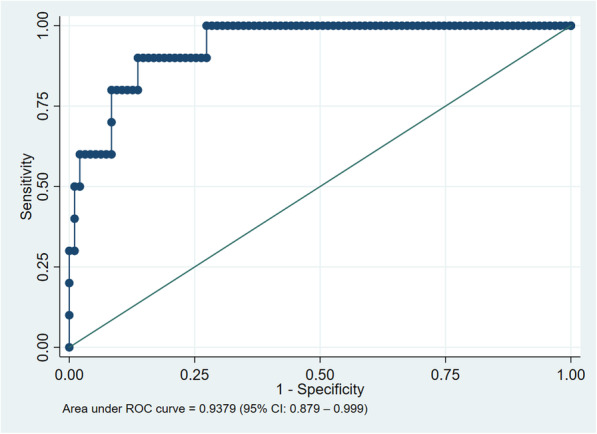
Reciever operating characteristics curve of mid-upper arm circumference based on BMI < 18.5 kg/m^2^, only males

ROC curves were generated for all participants (Fig. 2), and separately for women (Fig. 3) and men (Fig. 4). There was no difference between men and women. AUROCC for MUAC were high (> 0.90) for all groups as illustrated in Figs. 2, 3 and 4.

A comparison of SENS, SPEC, PPV, NPV and YI of MUAC cutoffs for every 0.5 cm ranging from 20 to 37.5 cm is presented in Table 2. YI revealed an optimal statistically-derived MUAC cutoff of 24.5 cm (YI = 0.75; sensitivity 92.9; specificity 82.5).

**Table 2:** Measures of diagnostic accuracy, comparison of MUAC cutoffs for every 0.5 cm

MUAC cutoff (cm)	Sensitivity (SENS)	Specificity (SPEC)	Positive Predictive Value (PPV)	Negative Predictive Value (NPV)	Youden’s Index (YI)
< 20	3.6	100	1	0.91	0.04
< 20.5	7.1	100	1	0.92	0.07
< 21	17.9	100	0.86	0.93	0.18
< 21.5	21.4	99.6	0.85	0.94	0.21
< 22	39.3	99.3	0.54	0.95	0.39
< 22.5	46.4	96.0	0.53	0.96	0.42
< 23	64.3	94.2	0.47	0.97	0.58
< 23.5	71.4	91.6	0.39	0.98	0.63
< 24	85.7	86.5	0.35	0.99	0.72
< 24.5	92.9	82.5	0.30	0.99	0.75
< 25	96.4	77.4	0.26	0.99	0.74
< 25.5	96.4	71.9	0.23	1	0.68
< 26	100	65.3	0.19	1	0.68
< 26.5	100	57.3	0.17	1	0.57
< 27	100	51.5	0.16	1	0.51
< 27.5	100	46.4	0.14	1	0.46
< 28	100	39.1	0.13	1	0.39
< 28.5	100	35.4	0.12	1	0.35
< 29	100	28.8	0.12	1	0.29
< 29.5	100	26.3	0.11	1	0.26
< 30	100	22.6	0.11	1	0.23
< 30.5	100	19.3	0.10	1	0.19
< 31	100	15.7	0.10	1	0.16
< 31.5	100	11.7	0.10	1	0.12
< 32	100	9.1	0.10	1	0.09
< 33.5	100	7.7	0.10	1	0.08
< 34	100	5.5	0.10	1	0.05
< 34.5	100	4.4	0.09	1	0.04
< 35	100	2.9	0.09	1	0.03
< 35.5	100	2.2	0.09	1	0.02
< 36	100	1.8	0.09	1	0.02
< 36.5	100	1.1	0.09	1	0.01
< 37	100	0.7	0.09	1	0.01
> 37.5	100	0.00	0.09	1	0

Finally, Table 3 shows the results from the same analysis in sex- and disease-specific subgroups.

**Table 3:** Sensitivity and Specificity stratified by sex and rheumatic heart disease status

MUAC cutoff (cm)	Women		Men		Rheumatic Heart Diseases (RHD)		Non-Rheumatic Heart Disease (non-RHD)	
	SENS	SPEC	SENS	SPEC	SENS	SPEC	SENS	SPEC
< 20.0	5.6	100	0	100	0	100	7.1	100
< 20.5	11.1	100	0	100	0	100	14.3	100
< 21.0	22.2	100	10.0	100	14.3	100	21.4	100
< 21.5	22.2	99.4	20.0	100	14.3	98.7	28.6	100
< 22.0	50.0	98.9	20.0	100	42.9	98.7	35.7	99.5
< 22.5	55.6	94.4	30.0	99.0	42.9	90.8	50.0	98.0
< 23.0	72.2	92.2	50.0	97.9	64.3	89.5	64.3	96.0
< 23.5	77.8	88.3	60.0	97.9	71.4	85.5	71.4	93.9
< 24.0	88.9	83.8	80.0	91.6	78.6	81.6	92.9	88.4
< 24.5	100	79.9	80.0	87.4	92.9	75.0	92.9	85.4
< 25.0	100	76.5	90.0	79.0	100	67.1	92.9	81.3
< 25.5	100	71.5	90.0	72.6	100	61.8	92.9	75.8
< 26.0	100	63.7	100	68.4	100	52.6	100	70.2
< 26.5	100	57.0	100	57.9	100	42.1	100	63.1
< 27.0	100	53.1	100	48.4	100	38.2	100	56.6

## Discussion

This study found a strong correlation between BMI and MUAC in both male and non-pregnant female adults in Nepal, and a statistically-derived MUAC cutoff for an underweight equivalent to BMI < 18.5 kg/m^2^ of 24.5 cm with similar results in men and women. These findings suggest that MUAC could be used as a screening tool for adult underweight in a low-resource setting, the same way BMI is currently being used. Other studies have found similar correlations in adult populations [2, 7, 10–15] and with a high correlation across ethnicity and sex. While there is consensus about the usefulness of MUAC, the optimal cutoff to detect underweight is still not determined. The cutoff can be described by YI, and with this method, we found that a MUAC of 24.5 cm would be the cutoff best associated with a BMI < 18.5 kg/m^2^. Other studies also using YI found that the MUAC cutoff should be between 21.9–25.1 cm [7, 11, 13, 15, 16]. However, we believe the optimal cutoff should not be determined strictly based on an equation, but instead take into account the setting it is to be used in. Deciding on a cutoff is a balance between not wanting to miss any undernourished people (having a high sensitivity) and at the same time, not wanting to refer people not actually needing nutritional support (high specificity). Choosing a cutoff with low specificity could put additional strain on already pressured health systems in low-resource settings. However, we believe the consequences of missing someone in need of nutritional support who may very rarely seek health support, should outweigh the economic burden of low specificity. Accordingly, we emphasized the highest sensitivity possible while still maintaining an acceptably high specificity (in this case, at least 75 and most often > 80). Based on our analysis, we suggest a setting-based MUAC cutoff of 24.5 cm to be ideal (it has the highest possible SENS with a SPEC > 80, see Table 2). Consequently, both the statistically-derived and setting-based cutoff are identical in our study. In a large individual participant data meta-analysis by Tang et al. a MUAC cutoff of ≤24 cm was suggested [7], which corresponds well with our suggestion.

Concerns about sex-related difference in muscle mass and the possible impact this could have on a non-sex specific MUAC cutoff are often raised. To address this issue, we repeated our analysis in both sex- and disease-related subgroups (Table 3). For both men and women, MUAC cutoffs of < 24.0 cm and < 24.5 cm had the highest possible SENS with a SPEC > 80 (if rounded up for women). In both sexes, MUAC correlated highly with BMI and displayed very similar ROC curves, although slightly better in women. This is not necessarily an expression of higher accuracy amongst women but could simply be because we included almost twice as many women as men in our study. Because we observed such small sex-differences and for the sake of simplicity, we recommend using the same cutoff in both women and men, since the intention is to use MUAC measurement for screening a high number of people with the lowest possible complexity.

While our analysis is based on the current practice of underweight defined as BMI < 18.5 kg/m^2^, it is important to consider if this is the optimal standard in all people. The chronically ill could possibly benefit from a higher value as is suggested in a study on adult patients with HIV, which showed that a BMI > 25 kg/m^2^ was associated with improved treatment response to antiviral medicine and thus a better treatment outcome [17]. In our study population, a large proportion suffered from chronic cardiovascular disease. While a prevalence this high is uncommon in the general population, a high prevalence of various chronic diseases is possible. This is due to the double burden of disease seen in low- and middle-income countries, where emerging epidemics of non-communicable diseases spread while infectious diseases remain a public health concern [18, 19]. In these chronically ill patients, monitoring nutritional status is especially important because of their change in metabolism leading to increased catabolism, which puts them at higher risk of morbidities and eventually mortality [20]. In advanced chronic diseases, cachexia is a central trait that could be detected early on by measuring MUAC. A BMI above but close to 18.5 kg/m^2^ is still worrisome and should be dealt with, especially in settings where chronic diseases are highly prevalent. We thus find it reasonable to emphasize the importance of a high sensitivity when selecting a MUAC cutoff.

This study has some limitations. Firstly, we included a large proportion of people suffering from a chronic heart disease, which is not representative of the population of Nepal. We cannot reject that our inclusion method might have caused selection bias, causing overrepresentation of people with any type of disease, since all participants were recruited in a hospital setting. Furthermore, only 9.3% of our participants had BMI < 18.5 kg/m^2^. This could be due to both the setting, including only people who reach the bigger cities in the country, as well as a relatively small sample size. A higher sample size might have increased the prevalence of the outcome, which again could increase the strength of the analysis.

## Conclusion

MUAC is strongly correlated with BMI in chronically ill and healthy adults in Nepal. In our setting, we found that a MUAC of 24.5 cm is the optimal cutoff in both women and men to identify underweight defined as BMI < 18.5 kg/m^2^. Thus, with its low cost and simplistic nature, MUAC could be considered an alternative to BMI in detecting adult underweight, if BMI is not feasible in the given situation.

## Data Availability

The datasets used and/or analysed during the current study are available from the corresponding author on reasonable request.

## Abbreviations

AUROCC: Area under ROC curve
BMI: Body Mass Index
HIV: Human Immunodeficiency Virus
kg: kilogram
m: meter
MUAC: Mid-Upper Arm Circumference
NPV: Negative predictive value
PPV: Positive predictive value
RHD: Rheumatic heart disease
ROC: Receiver operating characteristics
SENS: Sensitivity
SPEC: Specificity
YI: Youden’s Index

## References

[1] Physical status: the use and interpretation of anthropometry (1995). Report of a WHO Expert Committee. World Health Organ Tech Rep Ser.

[2] James WP, Mascie-Taylor GC, Norgan NG, Bistrian BR, Shetty PS, Ferro-Luzzi A (1994). The value of arm circumference measurements in assessing chronic energy deficiency in third world adults. Eur J Clin Nutr.

[3] Immink MD, Flores R, Diaz EO (1992). Body mass index, body composition and the chronic energy deficiency classification of rural adult populations in Guatemala. Eur J Clin Nutr.

[4] Chen Y, Ge W, Parvez F, Bangalore S, Eunus M, Ahmed A (2014). A prospective study of arm circumference and risk of death in Bangladesh. Int J Epidemiol.

[5] Schaap LA, Quirke T, Wijnhoven HAH, Visser M (2018). Changes in body mass index and mid-upper arm circumference in relation to all-cause mortality in older adults. Clin Nutr.

[6] WHO Child Growth Standards and the Identification of Severe Acute Malnutrition in Infants and Children (2009). A Joint Statement by the World Health Organization and the United Nations Children's Fund.

[7] Tang MC AM, Dong K, Wanke C, Bahwere P, Bose K, Chakraborty R, Charlton K, Hong S, Nguyen P, Patsche CB, Deitchler M, Maalouf-Manasseh Z (2017). Determining a Global Mid-Upper Arm Circumference Cutoff to Assess Underweight in Adults (Men and Nonpregnant Women). Food and Nutrition Technical Assistance III Project (FANTA).

[8] Alice M, Tang KD, Dietchler M, Chung M, Maalouf-Manasseh Z, Tumilowicz A, Wanke C (2013). Use of Cutoffs for Mid-Upper Arm Circumference (MUAC) as an Indicator or Predictor of Nutritional and Health-Related Outcomes in Adolescents and Adults: A Systematic Review. Food and Nutrition Technical Assistance III Project (FANTA).

[9] Youden WJ (1950). Index for rating diagnostic tests. Cancer..

[10] Collins S (1996). Using middle upper arm circumference to assess severe adult malnutrition during famine. JAMA..

[11] Benitez Brito N, Suarez Llanos JP, Fuentes Ferrer M, Oliva Garcia JG, Delgado Brito I, Pereyra-Garcia Castro F (2016). Relationship between Mid-Upper Arm Circumference and Body Mass Index in Inpatients. PLoS One.

[12] Kumar P, Sinha R, Patil N, Kumar V. Relationship between mid-upper arm circumference and BMI for identifying maternal wasting and severe wasting: a cross-sectional assessment. Public Health Nutr. 2019:1–5. 10.1017/S1368980019000727 Epub 2019/05/16. PubMed PMID: 31084660. 10.1017/S1368980019000727 PMC10260639 31084660

[13] Sultana T, Karim MN, Ahmed T, Hossain MI (2015). Assessment of under nutrition of Bangladeshi adults using anthropometry: can body mass index be replaced by mid-upper-arm-circumference?. PLoS One.

[14] Bisai S, Bose K (2009). Undernutrition in the Kora Mudi tribal population, West Bengal, India: a comparison of body mass index and mid-upper-arm circumference. Food Nutr Bull.

[15] Das P, Khatun A, Bose K, Chakraborty R (2018). The validity of mid-upper arm circumference as an indicator of low BMI in population screening for undernutrition: a study among adult slum dwellers in eastern India. Public Health Nutr.

[16] Chakraborty R, Bose K, Koziel S (2011). Use of mid-upper arm circumference in determining undernutrition and illness in rural adult Oraon men of Gumla District, Jharkhand, India. Rural Remote Health.

[17] Koethe JR, Jenkins CA, Shepherd BE, Stinnette SE, Sterling TR (2011). An optimal body mass index range associated with improved immune reconstitution among HIV-infected adults initiating antiretroviral therapy. Clin Infect Dis.

[18] Bygbjerg IC (2012). Double burden of noncommunicable and infectious diseases in developing countries. Science..

[19] Boutayeb A (2006). The double burden of communicable and non-communicable diseases in developing countries. Trans R Soc Trop Med Hyg.

[20] Cederholm T, Barazzoni R, Austin P, Ballmer P, Biolo G, Bischoff SC (2017). ESPEN guidelines on definitions and terminology of clinical nutrition. Clin Nutr.

